# The usefulness, reliability, and quality of YouTube video clips on congenital muscular torticollis: A STROBE compliant study

**DOI:** 10.1097/MD.0000000000030502

**Published:** 2022-09-16

**Authors:** Kil-Yong Jeong, Hyun Jung Lee, Shin-Young Yim

**Affiliations:** a The Center for Torticollis, Department of Physical Medicine and Rehabilitation, Ajou University School of Medicine, Suwon, Republic of Korea; b Department of Physical Medicine and Rehabilitation, Jeju National University Hospital, Jeju Special Self-Governing Province, Republic of Korea.

**Keywords:** congenital torticollis, consumer health information, quality, reliability, social media, webcast

## Abstract

This study aimed to evaluate the usefulness, reliability, quality, and related characteristics of YouTube video clips on congenital muscular torticollis (CMT). This cross-sectional study analyzed 47 YouTube video clips on CMT. They were classified as either useful or misleading by 2 rehabilitation doctors. The modified DISCERN tool and the Global Quality Scale (GQS) were used to evaluate their reliability and quality. An analysis was conducted using the characteristics, such as presenters, ownership of YouTube channel accounts, countries, contents, and the video popularity. Of the 47 YouTube video clips, 8 (17%) were evaluated as misleading, which indicated that they included at least one scientifically unproven piece of information on CMT or more. They were less reliable and of lower quality than the useful video clips. The video clips presented by healthcare professionals were more useful compared to those presented by others (*P* = .015). However, the video popularity was not related to its usefulness. The reliability and quality (3.70 ± 0.82 vs 0.75 ± 0.50 and 2.95 ± 1.21 vs 1.50 ± 1.00) assessed by the modified DISCERN tool and GQS, respectively, were significantly higher in the video clips presented by healthcare professionals compared to those presented by others. There were misleading YouTube video clips on CMT. Video clips presented by healthcare professionals could be more useful, reliable, and of better quality. The popularity of the video clips does not indicate more usefulness, reliability, and better quality. YouTube viewers should be aware of these findings. We recommend that the viewers preferentially choose video clips on CMT presented by healthcare professionals, not by the video popularity.

## 1. Introduction

Congenital muscular torticollis (CMT) is the third most common musculoskeletal disease in newborns, after clubfoot and developmental dysplasia of the hip.^[1–3]^ It is characterized by tightness and/or thickening of the ipsilateral sternocleidomastoid muscle, thereby limiting the motion of the neck.^[4–7]^ Most patients with CMT have a favorable prognosis after physical therapy, which includes stretching.^[4,8]^

Due to the COVID-19 pandemic, face-to-face treatment was often limited, leading people to seek medical information on the Internet. Family members of patients with CMT frequently access medical information, including a home exercise program, on CMT online, such as on YouTube. YouTube is a popular video-sharing platform commonly visited by patients and healthcare professionals for medical information.^[9]^

According to YouTube’s algorithm, related video clips are displayed continuously according to the video’s popularity, such as the number of views and likes.^[10]^ However, the usefulness, reliability, and quality of YouTube video clips on CMT are not yet known.^[11]^ Therefore, which characteristics of these video clips indicate more useful, reliable, and better quality are also unknown. Many views and likes may not always guarantee useful, reliable, or better-quality video clips. Therefore, the purpose of this study was to evaluate the usefulness, reliability, quality, and related characteristics of YouTube video clips on CMT.

## 2. Methods

This was a cross-sectional study based on the strengthening the reporting of observational studies in epidemiology guidelines^[12]^ and the reporting followed the guidelines for reporting observational studies.^[13]^

Since this study did not include human participants, the requirement for obtaining informed consent was waived by the Institutional Review Board of Ajou University Medical Center as of July 22, 2021.

### 2.1. The screening process of video clips on CMT

We performed a comprehensive electronic search on CMT on YouTube (https://www.youtube.com/) using the following 5 keywords in Ajou University School of Medicine, Suwon, South Korea on July 9, 2021: “congenital muscular torticollis,” “muscular torticollis,” “torticollis,” “wry neck,” and “tilted neck.”

The sample size was calculated as 51 video clips using the G*Power version 3.1.9.7,^[14]^ according to Cohen effect size of 0.46^[15]^ from a previous study on YouTube video clips using the DISCERN tool, 80% power, and 0.05 error margin. Considering the possibility of a loss of video clips, 300 video clips were identified.

The eligibility criteria were: video clips on CMT, video clips with voice commentary or narration, video clips that did not require adult authentication, and non-case reports. The exclusion criteria were: video clips irrelevant to CMT, video clips not made or spoken in English, video clips without voice commentary or narration, video clips that required adult authentication, and case reports.

All video clips were searched and sorted by relevance, which was the default setting. After case reports were excluded, the top 300 video clips found in a continuous list were identified.

**Figure 1.**
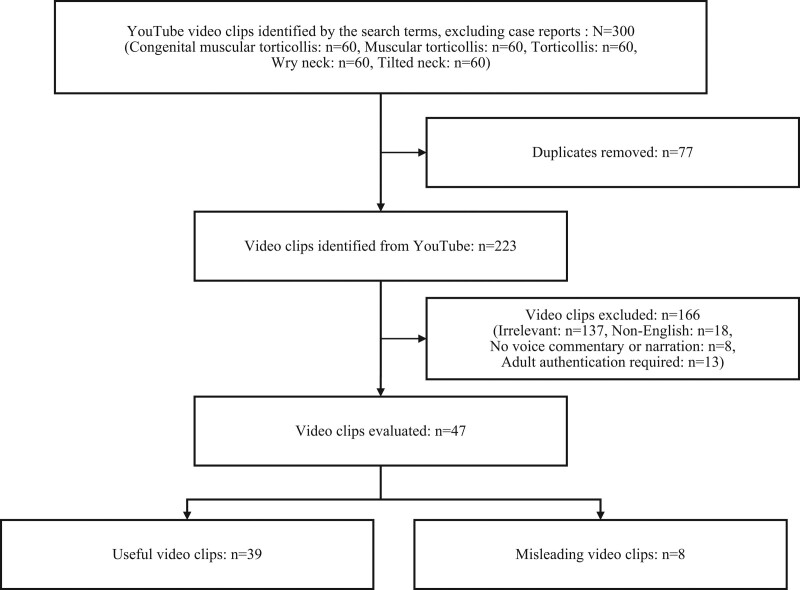
The screening process for the identification of video clips on congenital muscular torticollis.

Duplicated (n = 77), irrelevant (n = 137), non-English (n = 18), no voice commentary or narration (n = 8), or adult authentication required (n = 13) video clips were excluded (Fig. 1). Finally, 47 YouTube video clips on CMT were evaluated. A list of the 47 publicly available video clips used can be found in the Supplemental Digital Content 1, http://links.lww.com/MD/H267 along with the web addresses.

### 2.2. The evaluation of the characteristics of the video clips on CMT

The demographic characteristics of the video clips on CMT were collected regarding presenters, ownership of YouTube channel accounts, countries, and contents. The presenters were categorized into 3 classes: healthcare professionals (e.g., physical therapists, physicians, or occupational therapists), others, and unknowns, which meant that there were no specific details on the presenter. The ownership of YouTube channel accounts was classified by the owner as follows: healthcare information websites, hospitals, physicians, physical therapists, academic/professional organizations, or nonmedical individual YouTube creators. The countries of the owners were identified. The contents of the video clips were classified as follows: general information, physical therapy, diagnostic methods, surgery, treatment guidelines, and pathogenesis of CMT.

The characteristics of the video clips were obtained regarding the exposure period since the upload (days), duration of the video (minutes), number of views and likes, video popularity (views/day), number of likes per view, and number of likes per dislike. Video popularity was defined as the ratio of the number of views/days of the exposure period since the upload.

### 2.3. The assessment of the usefulness of the video clips on CMT

The usefulness of the video clips was assessed by 2 board-certified rehabilitation doctors (KYJ and HJL) with multiple years of experience related to pediatric and musculoskeletal diseases. Before the assessment, the 3 authors had consensus meetings. The video clips were grouped into useful or misleading video clips.^[9,16,17]^ Useful video clips meant they were thought to provide medically and scientifically accurate and useful information on CMT. Misleading video clips included those thought to contain at least one scientifically unproven piece of information on CMT or more. If the judgment of the 2 reviewers was different, the discrepancy was resolved by discussion between them.

### 2.4. The assessment of the reliability of the video clips on CMT

All video clips were independently assessed by 2 independent reviewers (KYJ and HJL). The reliability of the video clips was assessed using the modified DISCERN tool.^[16,18]^ The modified DISCERN tool was based on the DISCERN tool funded by the British Library.

It included 5 items and each question was answered with either “yes” (one point) or “no” (zero points):

1. Are the aims clear and achieved?
2. Are reliable sources of information used? (i.e., according to the publication cited and whether the speaker is a board-certified physical medicine and rehabilitation doctor).
3. Is the information provided balanced and unbiased?
4. Are additional sources of information listed for patient reference?
5. Does the video address areas of controversy/uncertainty?

In the modified DISCERN tool, zero indicated the poorest reliability, and 5 indicated the highest reliability.

### 2.5. The assessment of the quality of the video clips on CMT

The quality of the video clips was assessed using the Global Quality Scale (GQS)^[16,19]^ by 2 independent reviewers (KYJ and HJL). The GQS, a subjective measure of the quality of video clips, was rated on a 5-point Likert scale.

Each video clip was rated with a score of 1, 2, 3, 4, and 5. Each score was expressed as follows:

Score 1: Poor quality, poor flow of the video, most information missing, and not at all useful for patients.
Score 2: In general, poor quality and flow, while some information was provided, many important topics were missing, and very limited use for patients.
Score 3: Moderate quality, suboptimal flow, some important information was adequately discussed, however, others were poorly discussed, and somewhat useful for patients.
Score 4: Good quality and generally good flow, most of the relevant information was listed, however, some topics were not covered, and useful for patients.
Score 5: Excellent quality and flow, and very useful for patients.

Therefore, score 1 indicated poor quality and score 5 indicated excellent quality.

### 2.6. Statistical analysis

The intraclass correlation coefficient (ICC) was determined for the usefulness, reliability, and quality of the video clips on CMT as a measure of agreement between the 2 rehabilitation doctors who assessed them. Descriptive statistics, which included the presenters, ownership of YouTube channel accounts, countries, and contents, were presented as numbers and percentages for categorical variables. Variables of the video clips, such as exposure period since upload, playtime, and number of views, likes, and dislikes, were presented as means and standard deviations. Comparisons of the characteristics were performed using Fisher exact test and Kruskal-Wallis test for categorical and continuous data, respectively. Differences between groups were evaluated by subsequent post hoc analysis using multiple pairwise comparison tests with Bonferroni adjustments. The statistical significance was set at *P <* .05. Statistical analyses were performed using R version 4.1.2 (R Foundation for Statistical Computing, Vienna, Austria).

## 3. Results

### 3.1. The characteristics of the video clips on CMT

The demographic characteristics of the video clips on CMT are presented in Table 1. Among the presenters, physical therapists were the most common, with 25 (53.2%), followed by 10 physicians (21.3%) and 2 occupational therapists (4.3%). The physicians consisted of 5 orthopedic surgeons, 2 rehabilitation doctors, and 3 others. Aside from these, there were 2 broadcasters, one personal blogger, one physical therapy student, and 6 unknowns. Regarding the ownership of the YouTube channel accounts, healthcare information websites were the most common, with 14 (29.8%), followed by hospitals, physicians, and physical therapists with 11 (23.4%), 8 (17.0%), and 6 (12.8%), respectively. Of the countries, the United States accounted for the most, with 26 (55.3%), followed by India with 11 (23.4%) and the United Kingdom with 3 (6.4%). Regarding the content, general information on CMT was the most common, with 22 (46.8%), followed by physical therapy for CMT with 16 (34%).

**Table 1:** The demographic characteristics of the video clips on congenital muscular torticollis (n = 47).

Demographic characteristics	Number of video clips (%)
**Presenters**	
Healthcare professionals	37 (78.8%)
Physical therapists	25 (53.2%)
Physicians	10 (21.3%)
Orthopedic surgeons	5 (10.6%)
Rehabilitation doctors	2 (4.3%)
Dentists	1 (2.1%)
Pediatricians	1(2.1%)
Plastic surgeons	1(2.1%)
Occupational therapists	2 (4.3%)
Others	4 (8.5%)
Broadcasters	2 (4.3%)
Personal bloggers	1 (2.1%)
Physical therapy students	1 (2.1%)
Unknowns	6 (12.8%)
**Ownership of the YouTube channel accounts**	
Healthcare information websites	14 (29.8%)
Hospitals	11 (23.4%)
Physicians	8 (17.0%)
Physical therapists	6 (12.8%)
Academic/professional organizations	4 (8.5%)
Non-medical individual YouTube creators	4 (8.5%)
**Countries**	
United States	26 (55.3%)
India	11 (23.4%)
United Kingdom	3 (6.4%)
Canada	2 (4.3%)
South Korea	2 (4.3%)
Kuwait	1 (2.1%)
Philippines	1 (2.1%)
Russia	1 (2.1%)
**Content**	
General information on CMT	22 (46.8%)
Physical therapy for CMT	16 (34.0%)
Diagnostic method on CMT	3 (6.4%)
Surgery for CMT	2 (4.3%)
Treatment guideline for CMT	2 (4.3%)
Pathogenesis of CMT	2 (4.3%)

CMT = congenital muscular torticollis.

The characteristics of the video clips on CMT are shown in Table 2. The exposure period of video clips on CMT since upload was 1233.51 ± 1001.33 days. The video popularity was 20.62 ± 27.84 views/day, which meant that the video clips were viewed 20.62 times a day on average.

**Table 2:** The characteristics of the video clips on congenital muscular torticollis (n = 47).

Characteristics	Values (mean ± SD; range)
Exposure period since the upload (days)	1233.51 ± 1001.33 (64–3418)
Duration of the video clips (minutes)	6.52 ± 9.86 (0.62–63.05)
Number of Views	27703 ± 47800.07 (2–221206)
Number of Likes	121.74 ± 171.92 (0–747)
Number of Dislikes	8.64 ± 13.79 (0–56)
Video popularity (views/day)*	20.62 ± 27.84 (0.03–124.28)
Number of Likes per View	0.013 ± 0.020 (0–0.118)
Number of Dislikes per View	0.001 ± 0.001 (0–0.005)
Number of Likes per Dislike	21.09 ± 24.39 (5–121)

SD = standard deviation.

* Video popularity: Number of views/ days of exposure period since the upload.

### 3.2. Inter-rater agreement of the usefulness, reliability, and quality assessment of the video clips on CMT

There was no disagreement on the assessment of the usefulness of 47 video clips with an ICC of 1. The agreement of the modified DISCERN score was good with an ICC of 0.898 (95% confidence interval 0.816–0.943).^[20]^ Furthermore, the agreement of the GQS was excellent with an ICC of 0.938 (95% confidence interval 0.889–0.965).

### 3.3. The usefulness of the video clips on CMT

Of the 47 YouTube video clips on CMT, 39 (83%) were assessed as useful. Hence, 8 (17%) were assessed as misleading, which indicated that they included at least one scientifically unproven piece of information on the CMT or more.

### 3.4. The reliability of the video clips on CMT

The reliability score evaluated by the modified DISCERN was 2.91 ± 1.18 (Table 3), where 0 indicated the poorest reliability and 5 indicated the highest reliability of each video clip.

**Table 3:** The reliability and quality of the video clips on congenital muscular torticollis (n = 47).

Characteristics	Values (mean ± SD)
Reliability assessed by the modified DISCERN*	2.91 ± 1.18
Quality assessed by the GQS†	3.98 ± 0.87

GQS = Global Quality Scale, SD = standard deviation.

* Modified DISCERN, where the total score 0 indicates the poorest reliability and 5 indicates the highest reliability.

† GQS, where score 1 indicates poor quality and 5 indicates excellent quality.

Regarding each item of the modified DISCERN, 43 video clips (91.5%) were clear and achieved (DISCERN 1). Reliable sources with 40 (85.1%) (DISCERN 2) and balanced and unbiased information with 38 (80.9%) (DISCERN 3) were used among the total video clips. However, the number of additional sources of information listed for patient reference (DISCERN 4) was only 9 (19.1%), and video clips that mentioned areas of uncertainty (DISCERN 5) were 7 (14.9%).

### 3.5. The quality of the video clips on CMT

The quality of the video clips evaluated by the GQS score was 3.98 ± 0.87 (Table 3), where score 1 indicated poor quality and 5 indicated excellent quality.

Score 4 meant good quality and useful for patients and generally good flow meaning that most of the relevant information was listed, although some topics were not covered.

### 3.6. The comparison of the reliability and quality by the usefulness of the video clips on CMT

**Figure 2.**
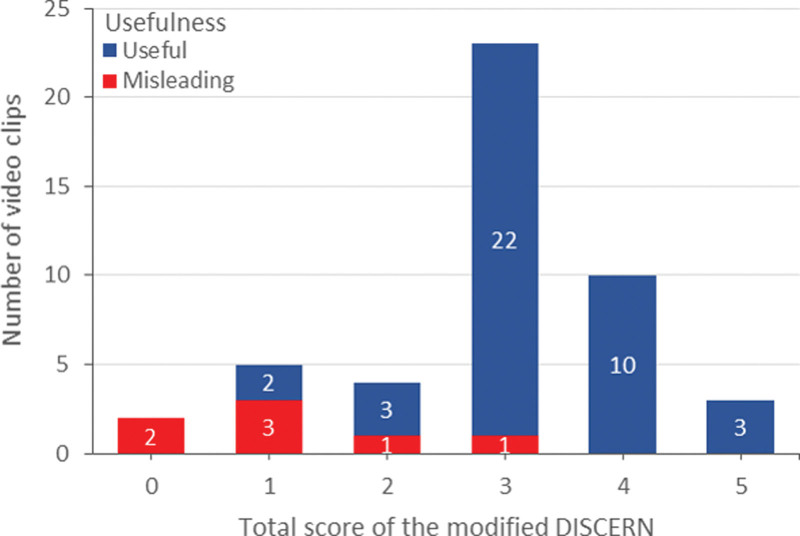
Distribution of the video clips by usefulness and the modified DISCERN scores.

**Figure 3.**
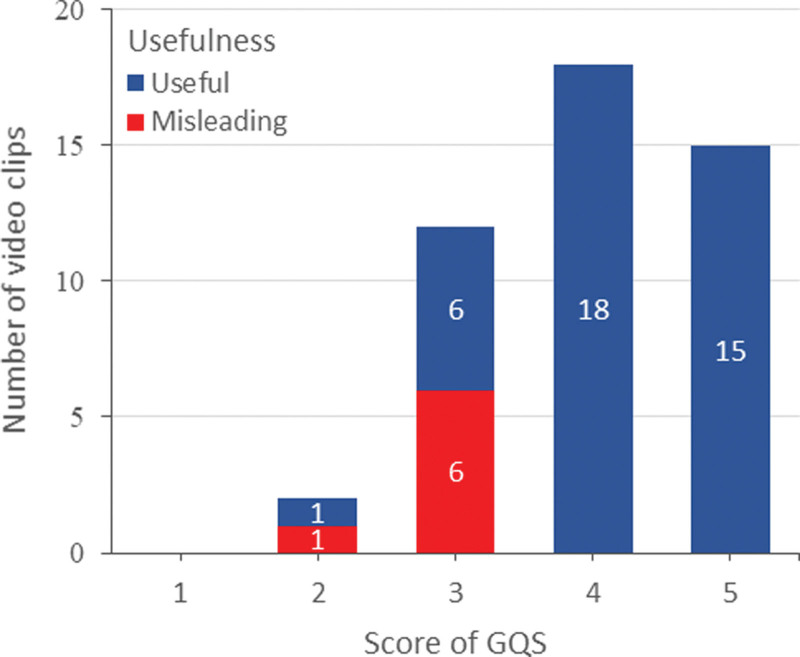
Distribution of the video clips by usefulness and the Global Quality Scale (GQS) score.

Table 4 shows a comparison of the modified DISCERN and GQS of video clips according to their usefulness. The modified DISCERN was significantly higher for the useful video clips compared to the misleading ones (3.28 ± 0.83 vs 1.13 ± 0.99, *P* < .001). The GQS was also significantly higher for the useful video clips compared to the misleading ones (4.23 ± 0.71 vs 2.75 ± 0.46, *P* < .001). The modified DISCERN scores ranged from 0 to 3 and 1 to 5 for the misleading and useful video clips, respectively (Fig. 2). The GQS score ranged from 2 to 3 and 2 to 5 for the misleading and useful video clips, respectively (Fig. 3).

**Table 4:** Comparison of the reliability and quality by the usefulness of the video clips on congenital muscular torticollis.

Characteristics	Useful video clips, n = 39 (83%)	Misleading video clips, n = 8 (17%)	*P*
Reliability assessed by the modified DISCERN*	3.28 ± 0.83	1.13 ± 0.99	<.001
Quality assessed by the GQS†	4.23 ± 0.71	2.75 ± 0.46	<.001

All values are mean ± standard deviation.

GQS = Global Quality Scale.

* Modified DISCERN, where the total score 0 indicates the poorest reliability and 5 indicates the highest reliability.

† GQS, where score 1 indicates poor quality and 5 indicates excellent quality.

### 3.7. The comparison of the characteristics by the usefulness of video clips on CMT

Table 5 shows a comparison of the demographic characteristics of the video clips according to their usefulness. There was a statistically significant difference in the usefulness of the video clips only by the presenters (*P* = .015). The subsequent post hoc analysis showed that the video clips presented by healthcare professionals were significantly more useful compared to those presented by others (*P* = .036).

**Table 5:** Comparison of the demographic characteristics by the usefulness of the video clips on congenital muscular torticollis.

Demographic characteristics	Useful video clips, n = 39 (83%)	Misleading video clips, n = 8 (17%)	*P*
**Presenters**			.015*
Healthcare professionals	33 (89.2%)	4 (10.8%)	
Physical therapists	21 (84.0%)	4 (16.0%)	
Physicians	10 (100%)	0 (0.0%)	
Occupational therapists	2 (100%)	0 (0.0%)	
Others	1 (25.0%)	3 (75.0%)	
Unknowns	5 (83.3%)	1 (16.7%)	
**Ownership of the YouTube channel accounts**			.196
Healthcare information websites	11 (78.6%)	3 (21.4%)	
Hospitals	8 (72.7%)	3 (27.3%)	
Physicians	8 (100%)	0 (0%)	
Physical therapists	6 (100%)	0 (0%)	
Academic/professional organizations	4 (100%)	0 (0%)	
Non-medical individual YouTube creators	2 (50.0%)	2 (50.0%)	
**Countries**			.636
United States	21 (80.8%)	5 (19.2%)	
India	9 (81.8%)	2 (18.2%)	
United Kingdom	3 (100%)	0 (0%)	
Canada	2 (100%)	0 (0%)	
South Korea	2 (100%)	0 (0%)	
Kuwait	1 (100%)	0 (0%)	
Philippines	0 (0%)	1 (100%)	
Russia	1 (100%)	0 (0%)	
**Content**			.687
General information on CMT	17 (77.3%)	5 (22.7%)	
Physical therapy for CMT	14 (87.5%)	2 (12.5%)	
Diagnostic method on CMT	3 (100%)	0 (0%)	
Surgery for CMT	1 (50.0%)	1 (50.0%)	
Treatment guideline for CMT	2 (100%)	0 (0%)	
Pathogenesis of CMT	2 (100%)	0 (0%)	

CMT = congenital muscular torticollis.

* The post hoc analysis reveals a significant difference between video clips presented by healthcare professionals and others (*P* = .036). There are no significant differences among physical therapists, physicians, and occupational therapists.

Table 6 shows a comparison of the characteristics of the video clips according to their usefulness. No characteristic of the video clips, which included video popularity, showed any significant difference regarding usefulness. The more popular video clips on CMT, characterized by more likes or views (*P* = .723) and more views a day (*P* = .729), were not more useful than the less popular ones.

**Table 6:** Comparison of the characteristics by the usefulness of the video clips on congenital muscular torticollis.

Characteristics	Useful video clips, n = 39 (83%)	Misleading video clips, n = 8 (17%)	*P*
Exposure period since the upload (d)	1195.39 ± 946.24	1419.38 ± 1296.45	.745
Duration of the video clips (min)	6.35 ± 10.03	7.34 ± 9.71	.988
Number of views	20512.59 ± 51434.67	18881.25 ± 23368.80	.835
Number of likes	131.49 ± 185.66	74.25 ± 63.25	.876
Number of dislikes	9.15 ± 14.93	6.13 ± 5.67	.764
Video popularity (views/d)^a^	22.40 ± 29.95	11.93 ± 10.88	.729
Number of likes per view	0.013 ± 0.020	0.016 ± 0.025	.723
Number of dislikes per view	0.001 ± 0.001	0.000 ± 0.000	.966
Number of likes per dislike	22.32 ± 26.38	15.15 ± 9.88	.654

All values are mean ± standard deviation.

a Number of views/day of exposure period since the upload.

### 3.8. The comparison of the reliability and quality by the demographic characteristics of the video clips on CMT

Table 7 shows a comparison of the modified DISCERN and GQS of the video clips according to their demographic characteristics. There was a statistically significant difference in the modified DISCERN score according to the presenter and channel account. post hoc analysis showed that the modified DISCERN and GQS scores of the video clips presented by healthcare professionals were significantly higher compared to those of the video clips presented by others (*P* = .005 for modified DISCERN, *P* = .027 for GQS). In addition, the modified DISCERN score of the video clips uploaded by physicians was significantly higher compared to that of the video clips uploaded by nonmedical individual creators (*P* = .043). Otherwise, there were no significant differences among physical therapists, physicians, and occupational therapists. Lastly, the GQS score of the video clips uploaded by physical therapists was significantly higher compared to that of the video clips uploaded by nonmedical individual creators (*P* = .029). There was no other significant difference among the YouTube channel account owners.

**Table 7:** Comparison of the reliability and quality by the demographic characteristics of the video clips on congenital muscular torticollis.

Demographic characteristics	Reliability*	*P*	Quality†	*P*
**Presenters**		.005‡		.024‡
Healthcare professionals	3.11 ± 0.99		4.14 ± 0.82	
Physical therapists	2.92 ± 1.00		4.08 ± 0.86	
Physicians	3.70 ± 0.82		4.20 ± 0.79	
Occupational therapists	2.50 ± 0.71		4.50 ± 0.71	
Others	0.75 ± 0.50		3.00 ± 0.00	
Unknowns	3.17 ± 1.17		3.67 ± 1.03	
**Ownership of the YouTube channel accounts**		.014§		.049∥
Healthcare information websites	2.86 ± 1.29		3.79 ± 0.89	
Hospitals	2.45 ± 1.09		4.00 ± 1.00	
Physicians	3.75 ± 0.89		4.25 ± 0.89	
Physical therapists	3.17 ± 0.41		4.67 ± 0.52	
Academic/professional organizations	3.75 ± 0.50		4.00 ± 0.00	
Non-medical individual YouTube creators	1.50 ± 1.00		3.00 ± 0.00	
**Countries**		.177		.171
United States	2.69 ± 1.19		4.04 ± 0.96	
India	3.09 ± 1.14		3.64 ± 0.58	
United Kingdom	3.33 ± 0.58		4.33 ± 0.58	
Canada	3.00 ± 0.00		4.00 ± 0.00	
South Korea	5.00 ± 0.00		5.00 ± 0.00	
Kuwait	3.00 ± 0.00		5.00 ± 0.00	
Philippines	1.00 ± 0.00		3.00 ± 0.00	
Russia	3.00 ± 0.00		3.00 ± 0.00	
**Content**		.056		.083
General information on CMT	2.82 ± 1.18		3.68 ± 0.84	
Physical therapy for CMT	2.75 ± 1.13		4.19 ± 0.91	
Diagnostic method on CMT	3.00 ± 0.00		4.67 ± 0.58	
Surgery for CMT	2.00 ± 1.41		3.50 ± 0.71	
Treatment guideline for CMT	4.00 ± 0.00		4.00 ± 0.00	
Pathogenesis of CMT	5.00 ± 0.00		5.00 ± 0.00	

CMT = congenital muscular torticollis.

* Reliability assessed by the modified DISCERN: where the total score 0 indicates the poorest reliability and 5 indicates the highest reliability.

† Quality assessed by the GQS: Global Quality Scale, where score 1 indicates poor quality and 5 indicates excellent quality.

‡ The post hoc analysis reveals a significant difference between the video clips presented by healthcare professionals and others (*P* = .005 for modified DISCERN, *P* = .027 for GQS). There are no significant differences among physical therapists, physicians, and occupational therapists.

§ The post hoc analysis reveals a significant difference between the video clips owned by physicians and nonmedical individual YouTube creators (*P* = .043). Otherwise, there are no significant differences.

∥ The post hoc analysis reveals a significant difference between the video clips owned by physical therapists and nonmedical individual YouTube creators (*P* = .029). Otherwise, there are no significant differences.

## 4. Discussion

This is the first study to evaluate the usefulness, reliability, quality, and related characteristics of YouTube video clips on CMT. CMT is one of the most common musculoskeletal diseases in newborns. Its prevalence ranges from 0.3% to 2.0% and can be as high as 3.92% in neonates.^[21–23]^ Most patients with CMT have a favorable prognosis after comprehensive physical therapy.^[24]^ However, patients with neglected CMT may have chronic pain, limited range of motion of the neck, and secondary musculoskeletal deformities, such as craniofacial asymmetry and scoliosis.^[25–27]^

Since most patients with CMT are newborns, the patient’s family caregivers need to understand the disease and pay attention to the appropriate treatment to improve the patient’s prognosis. Even though treatments by physical therapists may be more effective than a home program alone, parent or caregiver education and support to provide a daily home program are also useful for improving the patient’s prognosis.^[28]^

Recently, face-to-face treatment may be limited due to the COVID-19 pandemic. Hence, the obligations of family caregivers and the necessity of home programs are further emphasized. As YouTube becomes more and more accessible, family caregivers often use YouTube video clips for easy access to medical information regarding certain diseases.^[11]^ However, previous research has shown that not all YouTube video clips are useful.^[9,11,15–17,29–31]^ To the best of our knowledge, the usefulness, reliability, and quality of YouTube video clips on CMT are still unknown. Therefore, we evaluated the usefulness, reliability, and quality of the video clips of CMT and analyzed which characteristics of video clips indicated more useful, reliable, and better quality.

According to the results of this study, 39 out of the 47 identified video clips (83%) were assessed as useful. Nevertheless, 8 (17%) were assessed as misleading and showed relatively low modified DISCERN and GQS scores, which meant they contained unproven or inaccurate information on CMT which could lead to potential harm. Therefore, in this study, we performed characteristic analyses to find a way to choose more useful, reliable, and better-quality video clips.

The most significantly different characteristic among video clips according to usefulness, reliability, and quality was the presenter among the demographic characteristics. Furthermore, as a result of pairwise multiple comparison tests between the presenters, video clips presented by healthcare professionals such as physical therapists, physicians, and occupational therapists were significantly more useful compared to those presented by others, which consisted of broadcasters, personal bloggers, and students. The reliability (*P* = .005) and quality (*P* = .024) of the video clips presented by healthcare professionals were also higher compared to those presented by others.

None of the video clips presented by physicians were assessed as misleading. Since 2 video clips were regarding pathophysiology and most others were medical lectures, the video clips presented by physicians may be more suitable for professionals than the general YouTube viewers. Physical therapists presented the most video clips and the highest number of useful video clips, despite there being 4 misleading video clips. There was no statistically significant difference in usefulness between the video clips presented by physical therapists and those presented by physicians.

YouTube usually shows video clips based on algorithms determined by the number of likes and views.^[9,10]^ However, there was no significant difference in the total number of views and likes, and video popularity between the useful and misleading video clips on CMT. Thus, it is recommended that YouTube viewers preferentially choose video clips on CMT presented by healthcare professionals, not by video popularity. In addition, we suggest that healthcare professionals should be actively engaged in the development of YouTube video clips on CMT to ensure that patients or their family caregivers are not harmed by exposure to misleading information.

Studies of YouTube video clips on other diseases also found that the participation of healthcare professionals was important. Koo et al reported that 29 out of 140 video clips on gout (20.7%) were considered misleading even though they had more views compared to useful video clips.^[29]^ Meanwhile, most of the useful video clips were presented by rheumatologists. Therefore, they emphasized that healthcare professionals should participate in the development of video clips to provide accurate medical information. Onder et al also reported that the video clips on gout posted by academic institutions, professional organizations, and physicians had higher modified DISCERN and GQS scores indicating higher reliability and quality.^[9]^ In addition to this, Singh et al reported that all video clips on rheumatoid arthritis posted by university channels and professional organizations provided useful information.^[16]^ Abedin et al identified that most of the useful video clips on diabetic foot care were uploaded by medical professionals,^[30]^ while Elangovan et al found the same result in video clips on spondyloarthritis.^[31]^ Remvig et al found that less than half of the video clips on allergic rhinitis presented by nonprofessionals were useful, even though they are more popular and more patient-friendly than video clips from professional sources.^[32]^ These findings may not be limited to YouTube. Tam et al evaluated video clips of urinary tract infections posted on TikTok as well as on YouTube.^[33]^ YouTube video clips were more useful and reliable compared to TikTok, and more presenters were medical professionals on YouTube video clips than those on TikTok.

The strengths of this study are as follows. First, the inter-rater agreements between the 2 rehabilitation doctors on the usefulness, reliability, and quality assessment of the video clips on CMT were good and excellent. This was thought to be because CMT is a disease for which a relatively clear diagnosis and treatment are known.^[24,34]^ Second, a comprehensive and quantitative evaluation was conducted on the characteristics of YouTube video clips on CMT. According to the results, it could be suggested to YouTube viewers to check the presenter as a criterion for preferentially choosing video clips, not video popularity. Third, as a cross-sectional study, we complied with the strengthening the reporting of observational studies in epidemiology guidelines.

This study also had some limitations. First, this study evaluated video clips on YouTube CMT only spoken in English. In the non-English-speaking population, there may be differences in YouTube’s characteristics due to differences in medical systems or accessibility to the YouTube platform.^[35]^ Second, the number of YouTube video clips on CMT was relatively small compared to video clips on other diseases with higher prevalence, such as rheumatoid arthritis or gout.^[16,29]^ Third, not only a usefulness assessment of video clips, the DISCERN and GQS evaluation tools, which were widely used to evaluate the reliability or quality of YouTube video clips, could be affected by reviewer bias. However, as mentioned above, the inter-rater agreements on the assessment of the video clips on CMT were good and excellent. Fourth, as a cross-sectional study, a causal relationship could not be determined.

## 5. Conclusion

Approximately 83% of YouTube video clips on CMT were useful. However, this study found some inaccuracies in the medical information provided. Hence, 17% were misleading video clips on CMT. Video clips on CMT presented by healthcare professionals could be more useful, reliable, and of better quality. The popularity of video clips does not indicate more usefulness, reliability, and better quality. YouTube viewers should be aware of these findings. We recommend that viewers preferentially choose YouTube video clips on CMT presented by healthcare professionals, not by video popularity. Furthermore, healthcare professionals are recommended to actively participate in the development of YouTube video clips on CMT.

## Author contributions

**Conceptualization:** Shin-Young Yim.

**Data curation:** Kil-Yong Jeong, Hyun Jung Lee.

**Formal analysis:** Kil-Yong Jeong, Hyun Jung Lee, Shin-Young Yim.

**Investigation:** Kil-Yong Jeong, Hyun Jung Lee, Shin-Young Yim.

**Methodology:** Kil-Yong Jeong, Hyun Jung Lee, Shin-Young Yim.

**Project administration:** Shin-Young Yim.

**Resources:** Kil-Yong Jeong, Hyun Jung Lee, Shin-Young Yim.

**Software:** Kil-Yong Jeong, Hyun Jung Lee.

**Supervision:** Shin-Young Yim.

**Validation:** Kil-Yong Jeong, Hyun Jung Lee, Shin-Young Yim.

**Visualization:** Kil-Yong Jeong, Hyun Jung Lee.

**Writing – original draft:** Kil-Yong Jeong, Hyun Jung Lee, Shin-Young Yim.

**Writing – review & editing:** Kil-Yong Jeong, Hyun Jung Lee, Shin-Young Yim.

## Supplementary Material


