# Supplementary material for: The usefulness, reliability, and quality of YouTube video clips on congenital muscular torticollis: A STROBE compliant study
Source: Medicine (Baltimore). 2022 Sep 16;101(37):e30502. doi: 10.1097/MD.0000000000030502 (PMC9478286; doi:10.1097/MD.0000000000030502)
Supplement: Supplementary file 1 [file medi-101-e30502-s001.pdf]

Supplemental Digital Content. The 47 video clips on congenital muscular torticollis (CMT) used in this study.

No.	Title	Upload date	Duration (Minutes:Seconds)	Number of views	Number of likes
1	A new treatment for muscular torticollis	2013. 06. 07	9:06	20318	92
2	A quick and easy guide to baby torticollis.	2021. 02. 28	2:00	4221	27
3	Acute torticollis in kids wry neck twisted neck	2019. 11. 29	6:28	3856	70
4	At home stretches for infant with head tilt or tightness	2020. 04. 02	4:37	12210	195
5	Baby development torticollis neck rotation supine stretch	2020. 07. 30	0:45	17424	85
6	Baby development torticollis neck side-flexion supine stretch	2020. 07. 31	0:37	1997	8
7	Baby torticollis exercise	2013. 09. 14	1:05	60169	133
8	Congenital muscular torticollis	2018. 12. 07	5:51	638	15
9	Congenital muscular torticollis	2020. 04. 22	7:03	389	8
10	Congenital muscular torticollis physical therapy guideline evidence base	2018. 06. 29	4:48	14835	121
11	Congenital muscular torticollis Dr. Mallinath Gidaganti	2021. 05. 06	2:25	2	0
12	Congenital torticollis stretches	2014. 05. 25	1:47	11684	37
13	Easy at home infant torticollis exercise and physical therapy	2019. 06. 21	4:25	93088	747
14	Flat head syndrome and torticollis	2013. 09. 26	4:23	77387	331

15	Have concerns about torticollis?	2015. 03. 14	2:59	19341	40
16	How to prevent torticollis & plagiocephaly	2015. 01. 23	3:03	62662	237
17	Identifying torticollis	2015. 05. 07	2:44	140637	405
18	Left congenital torticollis	2018. 06. 28	1:08	412	3
19	Left torticollis home program	2015. 06. 01	3:04	9948	101
20	POC for congenital muscular torticollis	2021. 03. 17	30:13	13	1
21	Quick PT exercises for baby with right torticollis	2016. 10. 11	2:43	11915	462
22	Right congenital torticollis	2018. 06. 28	1:16	804	5
23	Stretch it out: How physical therapy helped with torticollis	2020. 09. 01	2:41	10207	44
24	Tilted neck congenital torticollis congenital disorder	2020. 07. 17	6:44	10096	95
25	Tips for torticollis stretching	2019. 11. 20	6:40	5924	37
26	Torticollis	2020. 01. 17	12:34	17870	86
27	Torticollis	2019. 11. 21	2:35	629	2
28	Torticollis	2020. 03. 23	17:40	803	12
29	Torticollis (Infant repositioning for plagiocephaly)	2020. 04. 18	8:44	6500	30
30	Torticollis (Infant)	2018. 01. 27	6:59	6559	0
31	Torticollis and the importance of tummy time	2012. 02. 29	4:24	221206	0

32	Torticollis breakdown	2013. 04. 15	2:57	49579	89
33	Torticollis exercises	2020. 04. 07	7:32	8439	203
34	Torticollis explained	2018. 01. 24	1:56	118564	425
35	Torticollis holding positions	2016. 03. 02	0:47	16064	83
36	Torticollis home exercises for infants	2020. 08. 14	3:25	28882	226
37	Torticollis operation under cervical plexus block	2020. 03. 10	5:40	7042	67
38	Torticollis quick screening guide	2014. 06. 19	1:45	170456	695
39	Torticollis CMT congenital muscular torticollis paediatric orthopaedics	2020. 10. 14	3:21	51	6
40	Torticollis Dr. Vivek Rege	2012. 05. 07	7:55	22294	80
41	Torticollis Part 1	2020. 06. 29	13:51	689	15
42	Torticollis wry neck & physiotherapy management	2018. 01. 10	4:34	4294	66
43	Torticollis wry neck: Anatomy lecture	2020. 12. 17	9:17	1388	48
44	Uptake of the congenital muscular torticollis guidelines	2018. 02. 07	3:14	497	4
45	Webinar: Practice pearls for the physiotherapy management of torticollis	2019. 05. 22	63:03	866	18
46	What is torticollis? What are its causes & symptoms?	2016. 12. 31	3:25	16927	88
47	What is torticollis? Wry neck? Causes diagnosis symptoms management	2018. 06. 25	5:18	12265	180
